# Bio-Based Poly(Ether Imide)s from Isohexide-Derived Isomeric Dianhydrides

**DOI:** 10.3390/polym9110569

**Published:** 2017-11-03

**Authors:** Xiaodong Ji, Zikun Wang, Zhen Wang, Jingling Yan

**Affiliations:** 1 Laboratory of Polymer Composites Engineering, Changchun Institute of Applied Chemistry, Chinese Academy of Science, Changchun 130022, China; jixiaodong@nimte.ac.cn (X.J.); wzkun@ciac.ac.cn (Zi.W.); wangzhen@ciac.ac.cn (Zh.W.); 2 State Key Laboratory of Polymer Physics and Chemistry, Changchun Institute of Applied Chemistry, Chinese Academy of Science, Changchun 130022, China

**Keywords:** isohexides, isomeric dianhydrides, poly(ether imide)s

## Abstract

In this work, four isohexide-derived isomeric dianhydrides were synthesized through a four-step procedure using isohexide and chloro-*N*-phenylphthalimides as the starting materials. The one-step solution polymerization of these dianhydrides with petroleum- or bio-based diamines enabled the synthesis of poly(ether imide)s (PEIs), which had viscosities of 0.41 to 2.40 dL∙g^−1^. The isohexide-derived PEIs were characterized based upon their solubility and their thermal, mechanical, and optical properties. The results showed that most of the isohexide-derived PEIs possessed comparable glass transition temperatures (*T*_g_), tensile strengths, and moduli to petroleum-based PEIs. However, the thermo-oxidative stability of the PEIs was found to be lower than that of the common petroleum-based PEIs. Moreover, the PEIs displayed good optical activity, which originated from their unique chiral isohexide moieties. The isomeric effects of dianhydride monomers on the properties of the resulting PEIs were comparatively studied. The results suggested that the corresponding 4,4′-linked PEIs possessed lower *T*_g_, higher mechanical properties, and higher specific rotations compared to 3,3′-linked polymers. Meanwhile, the polyimides with isomannide residue displayed higher *T*_g_ and more specific rotations than the corresponding polymers with isosorbide residue. These results contributed to more restricted rotations of phthalimide segments in 3,3′-linked or isomannide containing polyimides.

## 1. Introduction

Due to the depletion of petroleum reserves and the increase in ecological concerns caused by the production of vast amounts of non-degradable plastics, the development of polymers from renewable feedstocks has garnered considerable interest from both academia and industry [1,2,3]. In this regard, 1,4:3,6-dianhydrohexitols (isohexides) have been considered a family of attractive bio-sourced monomers for the polymer industry, owing to their several inherent advantages. Isohexides are rigid, chiral, nontoxic diols, which can be prepared with high purity using starch or cellulose as the raw materials. There are three isomers of isohexides, namely isosorbide (*exo-endo* isomer), isomannide (*endo-endo* isomer), and isoidide (*exo-exo* isomer) [4,5,6]. Among these isomers, Roquette is producing isosorbide at a rate of 20,000 tons per year [7]. In addition, isohexides have been used as bifunctional monomers for the synthesis of polycarbonate, polyester, polyurethane, and polyether through step-growth polymerization. The incorporation of isohexides into polymer backbones can result in achieving favorable properties, including high thermal stability, high glass transition temperature (*T*_g_), and improved optical transparency and activity [8,9,10,11,12,13,14,15,16]. Furthermore, Mitsubishi has commercialized isosorbide-based polycarbonate under the brand name of Durabio^®^, which has already found applications in automobile and electronic industries [13,14]. However, the low reactivity of sterically hindered, secondary hydroxyl groups in isohexides leads to polymers that have inadequate molecular weights and consequently possess inferior thermal and mechanical properties that seriously hamper their commercial applications [6]. Therefore, various isohexide derivatives were prepared to enhance their reactivity. In this regard, more reactive functional groups (such as vinyl, epoxy, amine, primary alcohols, carboxylic acids, and isocyanate) were introduced through either direct displacement or chain extension on the original secondary hydroxyl groups [17,18,19,20,21,22,23,24,25,26,27,28,29,30,31,32,33,34]. Consequently, high-molecular-weight polymers, with diverse backbone structures, were prepared through step-growth or chain-growth polymerizations and were evaluated for potential applications as commodity, performance, or functional polymeric materials [17,18,19,20,21,22,23,24,25,26,27,28,29,30,31,32,33,34].

Polyimides play an indispensable role in the aerospace, transportation, electronics, and automobile industries due to their high glass transition temperatures, high thermal stability, good chemical resistance, and remarkable mechanical and electrical properties [35,36,37,38,39]. The majority of dianhydride and diamine monomers, used for the synthesis of polyimides, are produced using petroleum-based chemicals as the raw materials. Furthermore, some of these monomers and their intermediates are highly toxic, carcinogenic, or endocrine-disrupting [40]. Therefore, considerable efforts have been devoted to develop dianhydrides, diamines, and polyimides using non-toxic bio-renewable feedstocks through different synthesis routes. Kaneko et al. prepared bio-based aromatic diamine 4,4′-diamino-α-truxilic acid (4ATA) through the photo-dimerization of 4-aminocinnamic acid (4ACA), which is the compound obtained from genetically manipulated *E. coli*. 4ATA-based polyimides displayed high thermal and mechanical properties, favorable optical transparency, and good cell compatibility [41]. Additionally, 4,4′-diaminostilbene and its reduced counterpart were also synthesized through Grubb’s metathesis reaction of 4ACA, and the resultant polyimides were found to possess good mechanical properties [42]. Yang and coworkers described the synthesis and characterization of polyimides using biomass-derived adenine as the diamine monomer and found that the corresponding polyimides exhibited outstanding thermal, mechanical, and dielectric properties [43]. Wadagaonkar et al. used cardanol-based diamine or diisocyanate to prepare polyimides and found that the cardanol-derived polyimide exhibited inferior thermal properties due to the presence of flexible aliphatic chains [44].

It is well known that isohexides are ideal building blocks for the synthesis of polyimides due to their inherent rigidity and feasible purification processes. Recently, a series of bio-based polyimides were synthesized using diamino-isosorbide, diamino-isoidide, bis(4-aminophenyl) isosorbide, and bis(4-aminophenyl) isomannide. These polyimides showed excellent optical transparency and optical activity, comparable thermal and mechanical properties to petroleum-based polyimides, and reasonably high bio-based contents [45,46]. Wang et al. incorporated trifluoromethyl moiety into isohexide-derived diamines and prepared polymers with high optical transparency and reduced water uptake [47]. Chen and coworkers reported the synthesis of bio-based dianhydride using isomannide and 4-nitrophthalonitrile as the raw materials and the preparation of isomannide-derived diamines through either direct substitution or chain extension reactions. Furthermore, a conventional two-step method has been used to prepare bio-based polyimides with good optical transparency and integrated properties using these isomannide-derived monomers [48].

In this work, the synthesis and characterization of bio-based poly(ether imide)s (PEIs) derived from isomeric bis(dicarboxyphenyl) isohexide dianhydrides have been reported. In addition, four isohexide-derived isomeric dianhydride monomers, three of which were reported for the first time, were prepared through the nucleophilic substitution reactions between *N*-phenyl-chlorophthalimides and isohexides, followed by hydrolysis, acidification, and dehydration reactions. The properties of synthesized bio-based PEIs were characterized in detail, and the isomeric effects of dianhydride monomers on the properties of the resulting polymers were systematically investigated.

## 2. Experimental

### 2.1. Materials

3-chloropthalic anhydride and 4-chlorophthalic anhydride were purchased from Changchun Hipolyking Co., Ltd., Changchun, China. 4,4′-oxydianiline (ODA), *m*-phenylenediamine (*m*-PDA), and 4,4′-methylenedianiline (MDA) were obtained from Changzhou Sunlight Pharmaceutical Co., Ltd., Changzhou, China. All the other chemicals were obtained from J&K Scientific Ltd., Beijing, China, and were used as received unless specified otherwise. Furthermore, *N*,*N*-dimethylformamide (DMF) was dried over calcium hydride, distilled under reduced pressure, and then stored over 4 Å molecular sieves. Additionally, 2,5-diamino-2,5-dideoxy-1,4:3,6-dianhydrosorbitol (DAIS) and 2,5-diamino-2,5-dideoxy-1,4:3,6-dianhydroiditol (DAII) were prepared according to the methods reported in the literature [44]. 3-chloro-*N*-phenylphthalimide and 4-chloro-*N*-phenylphthalimide were synthesized by refluxing chlorophthalic anhydrides and aniline in acetic acid, followed by recrystallization from *N,N*-dimethylacetamide [49].

### 2.2. Synthesis of Monomers

#### 2.2.1. 1,4:3,6-Dianhydro-2,5-di-*O*-(3-(*N*-phenyl-phthalimido))-d-sorbitol 

Isosorbide (14.61 g; 0.10 mol) and DMF (100 mL) were taken in a 500 mL three-necked flask, which was equipped with a magnetic stirrer and a nitrogen inlet and outlet. The mixture was stirred at 0 °C for 15 min in a nitrogen environment. Sodium hydride (7.20 g; 0.30 mol) was added to the mixture at 0 °C while stirring, and the reaction mixture was heated to 80 °C and maintained at this temperature for 15 h. After cooling to room temperature, 3-chloro-*N*-phenylphthalimide (57.24 g; 0.22 mol) in DMF (50 mL) was added in a separate vessel. Then the reaction mixture was stirred at 120 °C for 25 h. Upon cooling, 500 mL of 0.1 N HCl was added to the mixture. The precipitate was collected by filtration and washed thrice with deionized (DI) water. The crude product was recrystallized from a mixture of DMF and ethanol (1:2 *v*/*v* ratio, respectively) to obtain the yellow powder (29.4 g; yield of 50%) of 1,4:3,6-dianhydro-2,5-di-*O*-(3-(*N*-phenyl-phthalimido))-d-sorbitol. The product had the following characteristics. Melting point (m.p.): 176–178 °C, [a]_D_: (−) 26° (c = 0.01 g∙dL^−1^, *N*-methyl-2-pyrrolidone (NMP)). ^1^H NMR (400 MHz, DMSO-*d*_6_): δ 7.84 (2H, dd), 7.65 (1H, d), 7.51 (7H, m), 7.42 (6H, t), 5.20 (3H, m), 4.66 (1H, d), 4.24 (1H, dd), 4.03 (3H, dt). ^13^C NMR (400 MHz, DMSO-*d*_6_): δ 166.3, 164.8, 155.3, 154.1, 136.7, 133.8, 131.8, 128.8, 127.8, 127.5, 120.9, 117.6, 116.0, 115.4, 56.0, 82.3, 81.0, 77.4, 72.5, 71.2.

#### 2.2.2. 1,4:3,6-Dianhydro-2,5-di-*O*-(2,3-dicarboxyphenyl)-d-sorbitol Dianhydride (3,3′-ISDPA)

1,4:3,6-dianhydro-2,5-di-*O*-(3-(*N*-phenyl-phthalimido))-d-sorbitol (14.72 g; 0.025 mol) and aqueous sodium hydroxide solution (5% *wt/wt* ratio; 160 g) were taken in a 500 mL three-necked flask, which was equipped with a magnetic stirrer, a condenser, and a nitrogen inlet and outlet. The reaction was refluxed for 20 h in a nitrogen environment. After cooling to room temperature, the brown solution was filtrated to remove the insoluble impurities. Then the solution was acidified using excess concentrated HCl. The resultant reddish solid was collected by filtration, purified by recrystalization from 1,4-dioxane, and dehydrated using acetic anhydride to obtain the white solids (7.64 g, yield: 70%) of 3,3′-ISDPA. The product was found to have the following characteristics. m.p. 258–260 °C, [a]_D_: (+) 41 (c = 0.01 g∙dL^−1^, NMP). ^1^H NMR (400 MHz, DMSO-*d*_6_): δ 7.95 (2H, m), 7.76 (1H, d), 7.64 (3H, m), 5.25 (3H, m), 4.67 (1H, d), 4.05 (4H, m). ^13^C NMR (100 MHz, DMSO-*d*_6_): δ 165.4, 162.7, 158.5, 157.2, 140.7, 135.6, 123.6, 119.7, 118.4, 88.0, 84.5, 83.4, 80.2, 74.6, 73.7.

#### 2.2.3. 1,4:3,6-Dianhydro-2,5-di-*O*-(2,3-dicarboxyphenyl)-d-mannitol Dianhydride (3,3′-IMDPA) 

3,3′-IMDPA was prepared according to the procedure followed for preparing 3,3′-ISDPA, except that isomannide was used as the staring material instead of isosorbide. The product was found to have the following characteristics. Yield: 38%, m.p. 298–300 °C, [a]_D_: (+) 222° (c = 0.01 g∙dL^−1^, NMP). ^1^H NMR (400 MHz, DMSO-*d*_6_): δ 7.93 (2H, t), 7.77 (2H, d), 7.62(2H, d), 5.20 (2H, d), 5.02 (2H, d), 4.08 (2H, dd), 3.96 (2H, dd). ^13^C NMR (100 MHz, DMSO-*d*_6_): δ 165.2, 162.7, 158.4, 140.7, 135.2, 123.5, 119.6, 118.5, 82.2, 79.7, 72.9.

#### 2.2.4. 1,4:3,6-Dianhydro-2,5-di-*O*-(3,4-dicarboxyphenyl)-d-sorbitol Dianhydride (4,4′-ISDPA)

4,4′-ISDPA was prepared according to the procedure followed for preparing 3,3′-ISDPA, except that 4-chloro-*N*-phenylphthalimide was used as the staring material instead of 3-chloro-*N*-phenylphthalimide. The product was found to have the following characteristics. Yield: 36%, m.p. 104–107 °C, [a]_D_: (+) 133° (c = 0.01 g∙dL^−1^, NMP). ^1^H NMR (400 MHz, DMSO-*d*_6_): δ 8.01 (2H, dd), 7.70 (1H, s), 7.61 (1H, s); 7.55 (2H, dd), 5.23 (3H, dd), 4.62 (1H, s), 4.02 (4H, m). ^13^C NMR (100 MHz, DMSO-*d*_6_): δ 164.3, 162.9, 162.6, 133.9, 127.3, 123.7, 123.0, 110.4, 85.7, 81.9, 81.1, 77.9, 72.4, 71.3.

#### 2.2.5. 1,4:3,6-Dianhydro-2,5-di-*O*-(3,4-dicarboxyphenyl)-d-mannitol Dianhydride (4,4′-IMDPA)

4,4′-IMDPA was prepared according to the procedure followed for preparing 3,3′-ISDPA, except that 4-chloro-*N*-phenylphthalimide and isomannide were used as the staring materials instead of 3-chloro-*N*-phenylphthalimide and isosorbide. The product was found to have the following characteristics. Yield: 39%, m.p. 100–102 °C, [a]_D_: (+) 298° (c = 0.01 g∙dL^−1^, NMP). ^1^H NMR (400 MHz, DMSO-*d*_6_): δ 7.98 (2H, d), 7.74 (2H, s), 7.57 (2H, d), 5.21 (2H, d), 5.07 (2H, d), 4.01 (2H, dd), 3.88 (2H, dd). ^13^C NMR (100 MHz, DMSO-*d*_6_): δ 166.4, 165.2, 164.8, 136.2, 129.3, 125.7, 125.3, 112.5, 82.4, 79.6, 72.9.

### 2.3. Synthesis of Polymers

In this work, the bio-based PEIs were prepared in *m*-cresol through the conventional one-step method. Equimolar amounts of monomers were used to synthesize all the polymers. The typical synthesis procedure was as follows. First, 3,3′-ISDPA (0.3887 g; 0.89 mmol), 4,4′-ODA (0.1776 g; 0.89 mmol), and *m*-cresol (4 mL) were mixed in a 50 mL three-necked flask, which was equipped with a mechanical stirrer and a gas inlet and outlet. The mixture was stirred at 80 °C for 6 h and then at 180 °C for 24 h. After cooling to 80 °C, the viscous solution was mixed with ethanol (20 mL). After the fibrous polymer was collected by filtration, Soxhlet was extracted using ethanol for 24 h and dried in vacuum at 250 °C to afford the off-white solid (0.5022 g; yield of 94%) of polyimide 3,3′-ISDPA/ODA. The product was found to have the following characteristics. ATR-FTIR (film, ν, cm^−1^): 2925 (aromatic C–H stretching), 2865 (alicyclic C–H stretching), 1780 (asym C=O stretching), 1720 (sym C=O stretching), 1380 (C–N stretching), 1090 (C–O–C stretching in isosorbide residue). ^1^H NMR (400 MHz, DMSO-*d*_6_): δ 8.35 to 8.45 (m, 2H), 8.0 to 8.25 (m, 8H), 7.73 to 7.82 (m, 4H), 5.71 to 5.89 (m, 3H), 5.25 to 5.33 (m, 1H), 4.57 to 4.65 (m, 3H).

### 2.4. Film Formation and Characterization

The PEI solution in *m*-cresol (12 wt %) was filtrated to remove the insoluble impurities, degassed in a vacuum, and cast onto a dry flat glass substrate. The wet film was dried in a conventional oven at 80 °C for 8 h and then in a vacuum oven at 100 °C for 2 h. Afterwards, the film was heated subsequently at 150 °C, 200 °C, and 250 °C every two hours to completely remove the solvent. The film was peeled off from the substrate by immersion in hot water.

Attenuated total reflectance Fourier transform infrared spectroscopy (ATR-FTIR) was performed on a Bio-Rad Digilab Division FTS-80 spectrometer (Bio-Rad Digilab Division, Hopkinton, MA, USA). ^13^C NMR and ^1^H NMR spectra were recorded on a Bruker DRX 400 spectrometer (Bruker, Billerica, MA, USA), which operated at 400 MHz and used CDCl_3_ or DMSO-*d*_6_ as the solvents and tetramethylsilane as the internal standard. Inherent viscosities were measured using an Ubbelohde viscometer, at the concentration of 0.5 g∙dL^−1^ in *m*-cresol at 30 °C. The melting points were determined using an XT-4 melting point apparatus (Beijing Taike Apparatus Inc., Beijing, China). Thermogravimetric analysis (TGA) was carried out using a Perkin-Elmer TGA 2 analyzer (Perkin-Elmer, Waltham, MA, USA) with the heating rate of 10 °C∙min^−1^ in air. Differential scanning calorimetry (DSC) was conducted using a Q2000 DSC (TA Instruments, New Castle, DE, USA) with the heating rate of 10 °C∙min^−1^ within the temperature range of 40 °C to 300 °C in q nitrogen environment. Dynamic mechanical thermal analysis (DMTA) was conducted using a Q800 DMTA (TA Instruments, New Castle, DE, USA) in tensile mode from room temperature to 350 °C, with a heating rate of 3 °C∙min^−1^ and a frequency of 1 Hz. The glass transition temperature (*T*_g_) was determined as the peak temperature in the tan *δ*-curve. The tensile properties were evaluated using an Instron material testing system (Model 5982, Instron, Norwood, MA, USA) with a cross-head speed of 2 mm∙min^−1^. Five samples were tested for each film, and the averages were used to analyze the data. UV-Visible spectra were obtained using a Shimadzu UV-2550 spectrometer (Shimadzu, Kyoto, Japan) in the transmittance mode with the wavelength range of 200 to 800 nm. Specific rotation was measured on a Perkin Elmer Polarimeter 341LC (Perkin-Elmer, Waltham, MA, USA) at 20 °C under 589 nm with the sodium lamp. *N*-methyl-2-pyrrolidone (NMP) was used as the solvent, with a concentration of 0.01 g∙dL^−1^.

## 3. Results and Discussion

### 3.1. Synthesis of Monomers

**Scheme 1:**
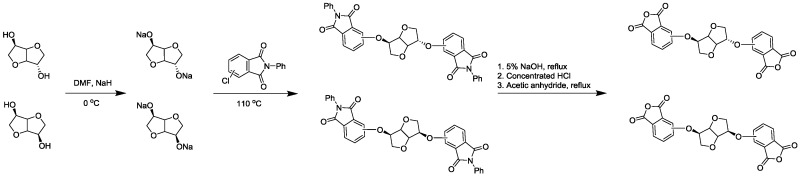
Synthetic routes for isohexide-derived isomeric dianhydrides.

Four isohexide-derived isomeric dianhydrides (3,3′-ISDPA, 3,3′-IMDPA, 4,4′-ISDPA, and 4,4′-IMDPA) were synthesized through a four-step procedure that comprised nucleophilic substitution between isohexides and chloro-*N*-phenylphthalimide, hydrolysis with NaOH, acidification using HCl, and dehydration in acetic anhydride (see Scheme 1). Chloro-*N*-phenylphthalimides were used as the starting materials instead of nitrophthalonitrile due to their low cost. The isolated yields for bis-(*N*-phenyl-phthalimido) isohexides were found to be moderate (around 50%), which can be attributed to low reactivity of the sterically hindered secondary hydroxyl groups present on isohexides. The yields for the hydrolysis, acidification, and dehydration reactions were relatively high (>80%), whereas the dianhydrides can be easily purified by recrystalization from acetic anhydride. The overall yields for isohexide-derived dianhydrides were found to be lying within the range of 30% to 40%.

**Figure 1:**
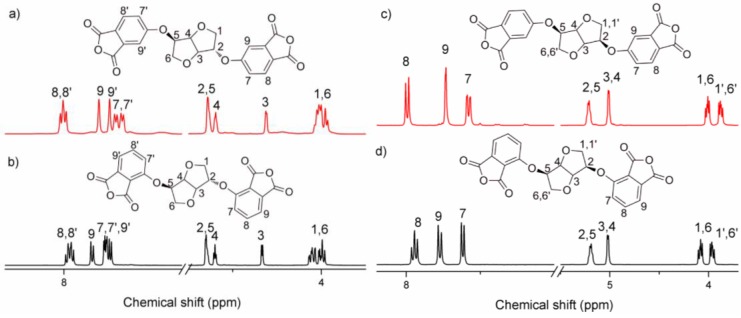
^1^H NMR spectra of isohexides-derived isomeric dianhydrides in DMSO-*d*_6_. (**a**): 4,4′-ISDPA; (**b**): 3,3′-ISDPA; (**c**): 4,4′-IMDPA; and (**d**): 3,3′-IMDPA.

**Figure 2:**
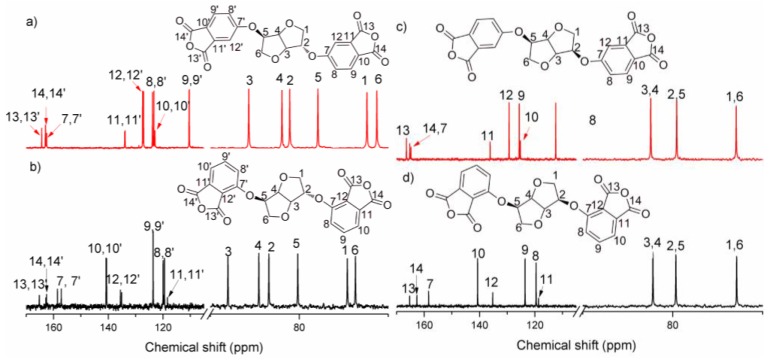
^13^C NMR spectra of isohexides-derived isomeric dianhydrides in DMSO-*d*_6_. (**a**): 4,4′-ISDPA; (**b**): 3,3′-ISDPA; (**c**): 4,4′-IMDPA; and (**d**): 3,3′-IMDPA.

The structures of dianhydrides were confirmed through the ^1^H and ^13^C NMR results, in which the signals for protons and carbons were fully assigned. As illustrated in Figure 1, the characteristic peaks at around 4.0 to 5.5 ppm and 7.5 to 8.0 ppm represent the protons in isohexides and in phthalic anhydride moieties, respectively. In ^13^C NMR spectra, the peaks at 70 to 90, 110 to 160, and 160 to 170 ppm were assigned to carbons in isohexides, benzene ring, and carbonyl groups, respectively (see Figure 2). Due to the asymmetrical structure of isosorbide moiety, the two phthalic anhydride segments in 4,4′-ISDPA and 3,3′-ISDPA were found to be configurationally different from each other and were reflected by two groups of signals for aromatic protons and carbons in NMR spectra. On the contrary, the two phthalic anhydride groups in 4,4′-IMDPA and 3,3′-IMDPA were found to be identical to each other. Therefore, only one group of signals for aromatic protons and carbons was observed in their NMR spectra. All dianhydride monomers were chiral with their specific rotation ([a]), their values being (+) 41°–(+) 298°. Furthermore, in comparison to 3,3′-linked dianhydrides, the 4,4′-linked dianhydrides exhibited higher specific rotation values for a given parent isohexide.

### 3.2. Synthesis of Polymers

**Scheme 2:**
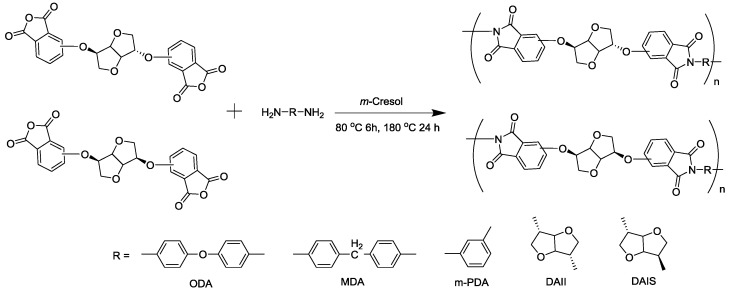
Synthesis of isohexide-derived PEIs.

Due to the formation of insoluble salt during the synthesis of poly (amic acid), alicyclic diamines are usually incompatible with the conventional two-step method. Therefore, in the present work, all PEIs were prepared in *m*-cresol through the conventional one-step method (see Scheme 2). The inherent viscosities of the resultant polymers were found to be lying within the range of 0.41 to 2.40 dL·g^−1^ (see Table 1), which was indicative of their moderate-to-high molecular weights. In addition, tough transparent films can be easily cast using the corresponding polyimide solutions, except for the polyimides 3,3′-IMDPA/*m*-PDA, 3,3′-IMDPA/DAII, and 3,3′-IMDPA/DAIS. The poor film-formability of 3,3′-IMDPA-based polyimides could be attributed to their rigid, twisted structures, which inhibited effective chain entanglements.

**Figure 3:**
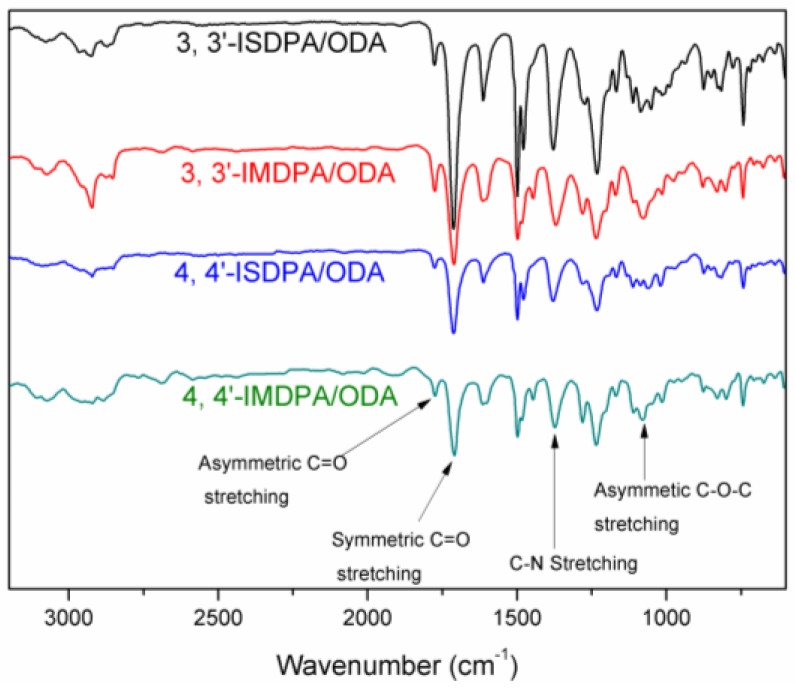
Representative FTIR spectra of isohexide-derived PEIs.

**Figure 4:**
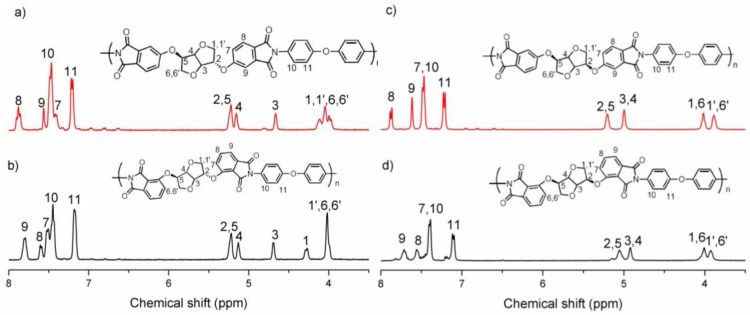
Representative ^1^H NMR spectra of isohexide-derived PEIs in DMSO-*d*_6_. (**a**): 4,4′-ISDPA/ODA; (**b**): 3,3′-ISDPA/ODA; (**c**): 4,4′-IMDPA/ODA; (**d**): 3,3′-IMDPA/ODA.

The structures of isohexide-derived PEIs were characterized through FTIR and ^1^H NMR techniques, whereas the corresponding spectra are compared in Figure 3 and Figure 4. In the FTIR spectra (see Figure 3), the absorption bands around 1780, 1720, and 1380 cm^−1^ were attributed to imide groups, while the peak at around 1090 cm^−1^ was related to C–O–C asymmetric stretching in isohexide moieties. Moreover, no peaks were detected for the characteristic absorption of amide groups (around 1650 cm^−1^), indicating that all the polymers were fully imidized. In the ^1^H NMR spectra (see Figure 4), the peaks at 7 to 8 ppm represented the aromatic protons in the phthalimide and ODA residues, whereas the signals between 3.5 and 5.5 ppm corresponded to the alicyclic protons in the isohexide segments. The results of FTIR and ^1^H NMR confirmed that the isohexide-derived PEIs with the proposed structures were successfully synthesized.

### 3.3. Properties of Bio-Based Poly(Ether Imide)s

#### 3.3.1. Bio-Based Contents and Solubility

In the present work, the bio-based content is defined as the percentage of organic carbon coming in the product from the bio-renewable feedstocks [48]. For bio-based PEIs, the carbons in isohexide segments were of bio-based origin, whereas the carbons in phthalimide and aromatic diamine residues were based on petrochemicals. As listed in Table 1, the bio-based contents for isohexide-derived polyimides were found to be lying within the range of 17.1–44.4%. In particular, the polyimides with isohexide moieties in both the dianhydride and the diamine residues possessed the bio-based content of 44.4%, which was markedly higher than the minimum requirement (25%) for bio-based materials suggested by USDA [50].

The solubility of isohexide-derived PEIs was tested in various solvents, and the results are presented in Table 1. All the polymers exhibited good solubility in *m*-cresol and NMP, while their solubility in low boiling solvents (chloroform and 1,4-dioxane) were found to be very limited. For a given dianhydride, PEIs based on DAII and DAIS showed lower solubility compared to those based on aromatic diamines. This can be ascribed to the incompatibility between the polar solvents and the nonpolar isohexide moieties. For certain diamines and catenation positions of phthalimide segments, the solubility of PEIs containing isosorbide residue was similar to that of the polyimides having isomannide residue. Moreover, 3,3′-linked polyimides showed higher solubility than 4,4′-linked polyimides due to their more bent structures, which loosened the chain packing, and hence increased the solubility.

#### 3.3.2. Thermal Properties

**Figure 5:**
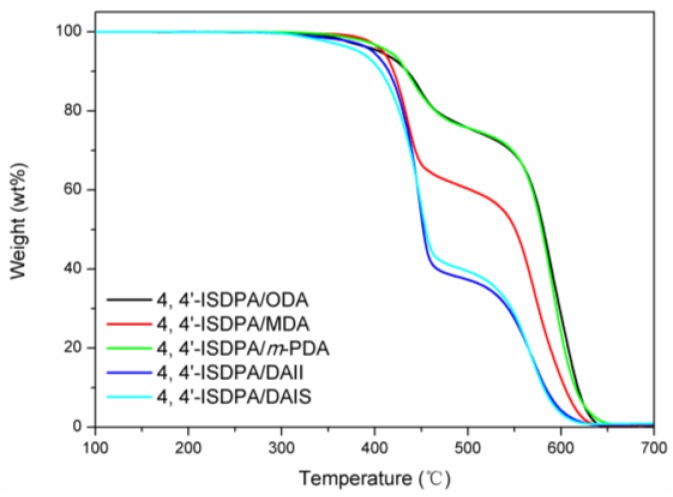
Representative TGA traces of isohexide-derived poly(ether imide)s (PEIs).

The thermo-oxidative stability of bio-based PEIs was evaluated using TGA in air. The results of the TGA analysis are presented in Table 2 and Figure 5. The 5% weight loss temperature (*T*_5%_) values of isohexide-derived PEIs were found to be lying within the range of 384–413 °C, which were lower than those of the petroleum-based PEIs (>440 °C) [38,51]. As illustrated in Figure 5, all the synthesized PEIs displayed a two-stage decomposition profile. The first weight loss at 350–450 °C originated form the degradation of alicyclic isohexide segments, while the second weight loss between 550 °C and 600 °C corresponded to the decomposition of aromatic contents. Furthermore, PEIs based on DAII and DAIS exhibited relatively lower thermo-oxidative stability than their aromatic diamines (ODA, MDA, and *m*-PDA) based counterparts, which could be due to their higher alicyclic contents. The structures of the dianhydrides did not have any significant impact on the degradation behavior of the resultant PEIs.

**Figure 6:**
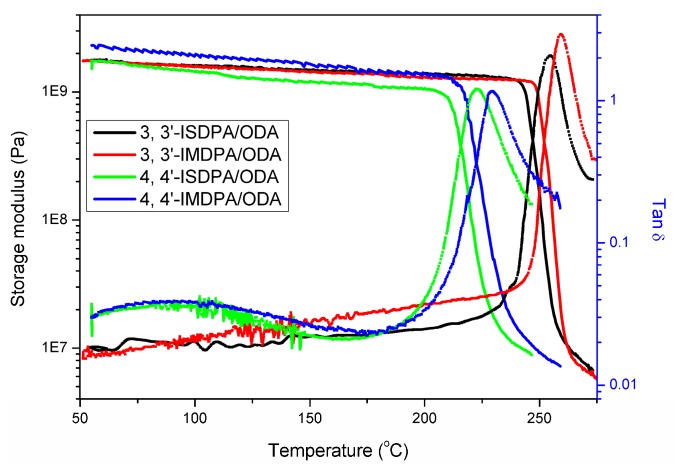
Representative DMTA curves of isohexides-derived PEIs.

The temperature dependence of storage modulus and tan *δ* for isohexide-derived PEIs was determined using DMTA. The corresponding DMTA curves are shown in Figure 6. The *T*_g_ values of the PEIs, which were determined as the peak temperatures of tan *δ* curves, ranged from 222 °C to 260 °C (see Table 2). The *T*_g_ values of polyimides were generally governed by the chain rigidity, intermolecular interactions, and rotational barrier of conformational changes, while the latter was considered as the most significant factor contributing to polyimides’ *T*_g_ values. The phthalimide segment acted as the ‘crank-shaft’, while the rotation of ‘crank-shaft’ around the ether bond led to the segmental motion of the polymer chains [35,38]. The conformational changes of thhe phthalimide segments in 3,3′-linked PEIs were more restricted than those in the 4,4′-linked PEIs, which were responsible for their higher *T*_g_ values. For certain diamine and isohexide structures, the *T*_g_ values of 3,3′-linked PEIs were found to be higher by 6 °C to 33 °C than those of the 4,4′-linked PEIs. Since the rotations were more pronounced in flexible polymer chains, the differences were larger when more flexible diamines were adopted. Furthermore, the β transition, which was broad but visible, was observed at 50 °C to 150 °C in the DMTA curves of 4,4′-linked PEIs, while no β transition was detected for 3,3′-linked PEIs. This phenomenon could also be attributed to the restricted rotation of 3-substituted phthalimide segments, which suppressed the local segmental relaxation at low temperatures.

The *endo* ether group on isohexide skeletons also imparted higher steric hindrance to the rotation of phthalimide segments. Therefore, the *T*_g_ values of isomannide-based PEIs were higher than those of the corresponding isosorbide-based PEIs. However, the differences were relatively smaller due to the flexibility of the ether bond. For a given dianhydride, PEIs derived from the ODA and MDA exhibited lower *T*_g_ values compared to those formed from *m*-PDA, DAII, and DAIS. This could be attributed to their flexible ether or methylene linkages. Additionally, DAII-based PEIs possessed higher *T*_g_ values than the DAIS-based PEIs due to their higher chain linearity, which stemmed from the *exo-exo* conformation of the diamine residue. The *T*_g_ values of isohexide-derived PEIs were also characterized using DSC, and the results were generally consistent with those of the DMTA (see Table 2).

#### 3.3.3. Mechanical Properties

**Figure 7:**
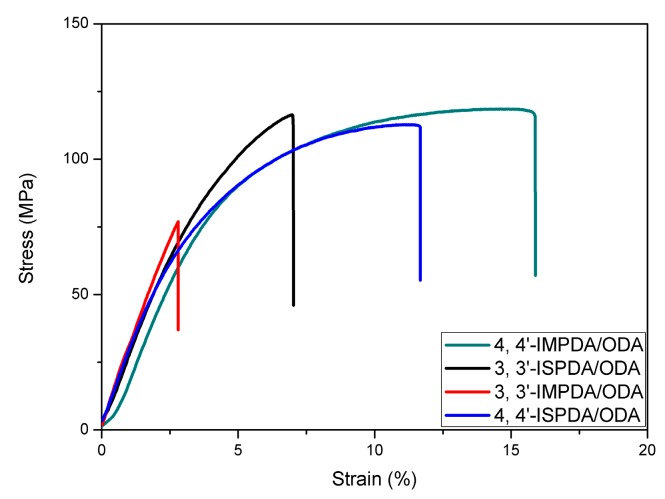
Representative stress-strain curves of isohexides-derived PEIs.

The mechanical properties of isohexide-derived PEIs were measured using tensile testing, and the results are summarized in Table 2 and Figure 7. The tensile strength, modulus, and elongation at break were found to be lying within the ranges of 80 to 132 MPa, 2.2 to 3.6 GPa, and 2.6% to 24.5%, respectively. The film toughness of 3,3′-linked PEIs was inferior to that of the 4,4′-linked PEIs, which can be attributed to their more bent structure, which suppressed effective chain entanglements. In particular, the 3,3′-linked polyimides displayed elongation at break values of less than 8%, while most of the 4,4′-linked polyimides showed elongation at break values of higher than 10%. A similar trend was also noted for tensile strength. Polyimides originating from 3,3′-IMPDA displayed the poorest mechanical properties because of their inefficient chain entanglement, which stemmed from their most twisted structures. For a given dianhydride, the moduli of polymers from *m*-PDA, DAII, and DAIS were generally higher than those of the polymers originating from ODA and MDA. This observation could be explained on the basis of the higher flexible linkage contents of the ODA- and MDA-based PEIs.

#### 3.3.4. Optical Properties

**Figure 8:**
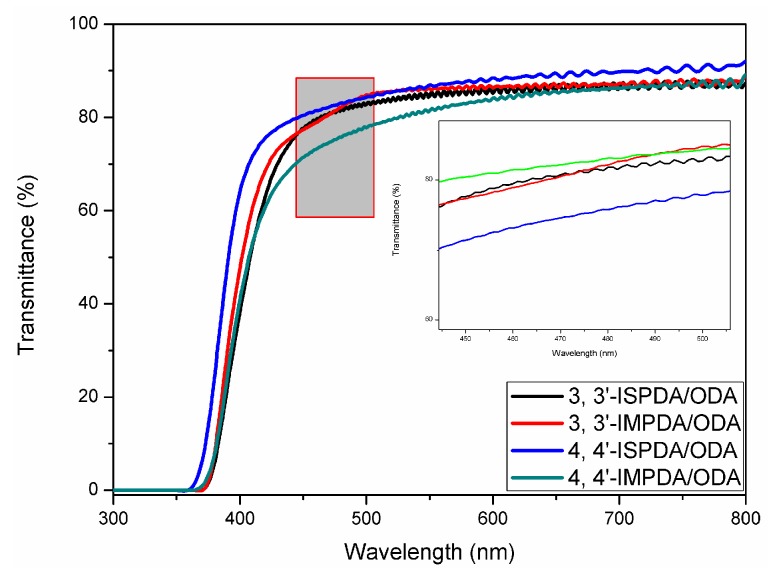
Representative UV-vis spectra of isohexides-derived PEIs (film thickness: 20 μm).

The optical properties of isohexide-derived polyimides were characterized using UV-Vis spectra measurements. The results are summarized in Table 3. The representative UV-Vis spectra of isohexide-derived PEIs are shown in Figure 8. The values of the cut-off wavelength (*λ*_0_) for these PEIs lay within the range of 363 to 378 nm. All the polyimides were colorless or pale yellow due to the incorporation of alicyclic isohexide moieties, which interrupted the electronic conjugation and suppressed the formation of inter- and intra-molecular interactions. The optical properties of the resultant polyimides were not significantly affected by the isomeric effects of the isohexide and phthalimide groups. The transmittance at 450 nm for isohexide-derived PEIs ranged from 61% to 80%.

All isohexide-derived PEIs were optically active due to the incorporation of chiral isohexide segments, while the values of specific rotation for these polymers and the corresponding model compounds are listed in Table 3. It is worth noticing that the PEIs and model compound based on 3,3′-ISDPA and aromatic diamines were levorotatory, with a specific rotation value of (−) 91°–(−) 100°, while 3,3′-ISDPA was dextrorotary, with a specific rotation value of (+) 41°. The reversal of specific rotations implied that some higher ordered structures may exist in these polymers. These results are consistent with those reported in the literature regarding various chiral polymers [52,53,54]. However, the polyimides 3,3′-ISDPA/DAII and 3,3′-ISDPA/DAIS were dextrorotary, with the incorporation of dextrorotary DAII or DAIS moieties. Except for the PEIs based on 3,3′-ISDPA and aromatic diamines, all other isohexide-derived PEIs were dextrorotary, with the specific rotation values lying within the range of (+) 86°–(+) 370°. The specific rotations of isohexide-derived PEIs were generally dictated by the structures and contents of optically active segments, as well as the linearity of the polymers. For a given diamine, polymers with isosorbide residues possessed lower specific rotations than those with isomannide residues because of the higher specific rotations of isosorbide moieties. Moreover, 3,3′-linked PEIs exhibited lower optical activity than the 4,4′-linked PEIs due to their more bent architectures. In addition, DAII- and DAIS-based polyimides exhibited the highest specific rotations among other PEIs for a given dianhydride. This was due to their higher isohexide contents. In general, the trends regarding the specific rotations of the model compounds agreed with those of their corresponding polyimides. The optical activity of isohexide-derived polyimides was impressive compared to other isohexide-derived polymers and chiral polyimides [52,55,56,57,58,59,60,61]. Isohexide-derived PEIs could be potentially used in the applications of chiral chromatographic separation of enantiomers, non-liner optical devices, and optical switches due to their combined properties of high *T*_g_, good chemical resistance, reasonably high optical transparency, and specific rotation.

## 4. Conclusions

In this study, four isohexide-derived isomeric dianhydrides were successfully prepared through the nucleophilic substitution reactions between isohexides and chloro-*N*-phenylphthalimides, followed by deprotection and dehydration reactions. Additionally, conventional one-step polycondensation of isohexide-derived dianhydrides with petroleum-based or bio-based diamines generated PEIs with a bio-based content of 17.7% to 44.4% and an inherent viscosity of 0.41 to 2.40 dg∙L^−1^. Owing to the inherent rigidity of the alicyclic isohexide segments, the isohexide-derived PEIs possessed *T*_g_ values and mechanical properties that were comparable to their petroleum-based counterparts. Moreover, isohexide-derived PEIs exhibited high optical activity due to their high isohexide contents, which was evidenced by their specific rotations, which lay within the range of (−) 16°–(+) 370°. In addition, the isomeric effects of isohexides and dianhydrides on the properties of resultant polymers were systematically investigated. The results showed that, in comparison to 3,3′-linked PEIs, the 4,4′-linked PEIs exhibited lower *T*_g_ values and solubility, more obvious β transition, and higher tensile strength, toughness, and specific rotations. Meanwhile, PEIs with isomannide residue displayed higher *T*_g_ values and more specific rotations than those with isosorbide residue. Owing to the excellent combination of high bio-based contents, impressive optical activity, and comparable thermal and mechanical properties, the isohexide-derived PEIs exhibit great potential for commercial use and can replace petroleum-derived PEIs. This work provided new insights into the isomeric effects of dianhydride monomers on the properties of the resultant polyimides, as well as into how to design and prepare bio-based polyimides with excellent properties and optical activity.

## Figures and Tables

**Table 1:** Bio-based contents, inherent viscosity, and solubility of isohexide-derived PEIs.

Polyimides	*η*_inh_ (dL·g^−1^) ^a^	Bio-Based content (%) ^b^	Solubility ^c^					
			*m*-Cresol	DMAc	CHCl_3_	NMP	DMSO	1,4-Dioxane
3,3′-ISDPA/ODA	0.69	17.6	++	++	-	++	+	+-
3,3′-ISDPA/MDA	0.56	17.1	++	++	+-	++	+-	+-
3,3′-ISDPA/*m*-PDA	0.54	21.4	++	+	-	++	+	-
3,3′-ISDPA/DAII	0.51	44.4	++	+-	-	++	+	+-
3,3′-ISDPA/DAIS	0.75	44.4	++	+-	+-	++	+	-
3,3′-IMDPA/ODA	1.50	17.6	++	++	-	++	++	-
3,3′-IMDPA/MDA	1.30	17.1	++	+	-	++	++	-
3,3′-IMDPA/*m*-PDA	- ^d^	21.4	+-	+	-	++	++	-
3,3′-IMDPA/DAII	2.40	44.4	++	-	-	-	-	-
3,3′-IMDPA/DAIS	0.72	44.4	++	+-	-	+-	-	-
4,4′-ISDPA/ODA	0.69	17.6	++	+	+-	+	+-	-
4,4′-ISDPA/MDA	0.59	17.1	++	+-	+-	++	+-	-
4,4′-ISDPA/*m*-PDA	0.46	21.4	++	+-	-	+	+	-
4,4′-ISDPA/DAII	0.41	44.4	++	+-	+-	++	+-	-
4,4′-ISDPA/DAIS	0.98	44.4	++	-	-	+-	+-	-
4,4′-IMDPA/ODA	0.72	17.6	++	-	-	+-	+-	-
4,4′-IMDPA/MDA	1.61	17.1	++	+	-	+	+	-
4,4′-IMDPA/*m*-PDA	0.46	21.4	++	+	-	++	+	-
4,4′-IMDPA/DAII	0.44	44.4	++	-	+-	++	+	-
4,4′-IMDPA/DAIS	0.76	44.4	++	-	+-	+	+	-

^a^ Measured in *m*-cresol at a concentration of 0.5 g∙dL^−1^ at 30 °C. ^b^ Calculated according to the method given in [50]. ^c^ Solubility was determined with 10 mg of polymers in 1 mL of solvent. ++: soluble at room temperature. +: soluble upon heating. +-: partially soluble or swelling. -: insoluble. DMAc: dimethylacetamide. DMSO: dimethyl sulfoxide. CHCl_3_: chloroform. ^d^ Not measured because it is only partially soluble in *m*-cresol.

**Table 2:** Thermal and mechanical properties of isohexide-derived PEIs.

Polyimides	*T*_g_^a^ (°C)	*T*_g_^b^ (°C)	*T*_5%_^c^ (°C)	Tensile strength (MPa)	Modulus (GPa)	Elongation at break (%)
3,3′-ISDPA/ODA	255	250	413	114	2.7	7.6
3,3′-ISDPA/MDA	247	247	404	101	2.5	6.8
3,3′-ISDPA/*m*-PDA	260	260	399	80	3.1	2.9
3,3′-ISDPA/DAII	255	249	384	80	3.6	2.6
3,3′-ISDPA/DAIS	253	255	388	106	3.2	6.3
3,3′-IMDPA/ODA	259	261	411	74	2.2	3.8
3,3′-IMDPA/MDA	252	254	406	85	2.2	4.3
3,3′-IMDPA/*m*-PDA	- ^d^	262	410	- ^d^	- ^d^	- ^d^
3,3′-IMDPA/DAII	- ^d^	262	399	- ^d^	- ^d^	- ^d^
3,3′-IMDPA/DAIS	- ^d^	259	393	- ^d^	- ^d^	- ^d^
4,4′-ISDPA/ODA	222	226	405	98	2.9	14.7
4,4′-ISDPA/MDA	230	227	409	111	2.7	13.4
4,4′-ISDPA/*m*-PDA	239	239	415	132	3.3	8.4
4,4′-ISDPA/DAII	243	249	398	117	3.2	8.3
4,4′-ISDPA/DAIS	239	239	384	129	3.5	12.6
4,4′-IMDPA/ODA	229	225	413	116	2.7	15.9
4,4′-IMDPA/MDA	246	251	402	115	3.0	24.5
4,4′-IMDPA/*m*-PDA	246	246	408	134	3.6	10.4
4,4′-IMDPA/DAII	245	244	393	116	3.4	11.1
4,4′-IMDPA/DAIS	242	239	386	119	3.5	9.3

^a^ Determined using DMTA with the heating rate of 3 °C∙min^−1^ at 1 Hz. ^b^ Determined using DSC under nitrogen environment with the heating rate of 10 °C∙min^−1^. ^c^ 5% Weight loss temperatures, measured using TGA in air with the heating rate of 10 °C∙min^−1^. ^d^ Not measured because the films were too brittle.

**Table 3:** Optical properties of isohexide-derived PEIs.

Polyimides ^a^	Specific rotation (°) ^b^	*λ*_0_ (nm) ^c^	*T*_450_ (%) ^d^
3,3′-ISDPA/ODA	(−) 100	373	78
3,3′-ISDPA/MDA	(−) 95	378	64
3,3′-ISDPA/*m*-PDA	(−) 91	377	61
3,3′-ISDPA/DAII	(+) 86	370	76
3,3′-ISDPA/DAIS	(+) 124	368	65
3,3′-IMDPA/ODA	(+) 230	373	77
3,3′-IMDPA/MDA	(+) 212	373	83
3,3′-IMDPA/*m*-PDA	(+) 258	365	-
4,4′-ISDPA/ODA	(+) 123	364	80
4,4′-ISDPA/MDA	(+) 123	373	63
4,4′-ISDPA/*m*-PDA	(+) 140	377	67
4,4′-ISDPA/DAII	(+) 203	369	78
4,4′-ISDPA/DAIS	(+) 248	365	72
4,4′-IMDPA/ODA	(+) 235	370	71
4,4′-IMDPA/MDA	(+) 233	363	80
4,4′-IMDPA/*m*-PDA	(+) 285	372	71
4,4′-IMDPA/DAII	(+) 344	369	66
4,4′-IMDPA/DAII	(+) 370	365	70
3,3′-ISDPA/Ph	(−) 26	-	-
3,3′-IMDPA/Ph	(+) 189	-	-
4,4′-ISDPA/Ph	(+) 121	-	-
4,4′-IMDPA/Ph	(+) 293	-	-

^a^ The optical properties of polyimides 3,3′-IMDPA/DAII and 3,3′-IMDPA/DAIS were not measured because of their poor film formability and limited solubility in NMP. 3,3′-ISDPA/Ph: 1,4:3,6-dianhydro-2,5-di-*O*-(3-(*N*-phenyl-phthalimido))-d-sorbitol. 4,4′-ISDPA/Ph: 1,4:3,6-dianhydro-2,5-di-*O*-(4-(*N*-phenyl-phthalimido))-d-sorbitol. 3,3′-IMDPA/Ph: 1,4:3,6-dianhydro-2,5-di-*O*-(3-(*N*-phenyl-phthalimido))-d-mannitol. 4,4′-IMDPA/Ph: 1,4:3,6-dianhydro-2,5-di-*O*-(4-(*N*-phenyl-phthalimido))-d-mannitol. ^b^ Measured at 20 °C in NMP with a concentration of 0.01 g∙dL^−1^. ^c^ Wavelength with a transmittance of <1%. ^d^ Transmittance at 450 nm.
